# Comparison of the course of multisystem inflammatory syndrome in children during different pandemic waves

**DOI:** 10.1007/s00431-022-04790-4

**Published:** 2023-01-31

**Authors:** Katarzyna Ptak, Izabela Szymońska, Anna Olchawa-Czech, Kornelia Kukla, Marta Cisowska, Przemko Kwinta

**Affiliations:** 1 Department of Pediatrics, Jagiellonian University Medical College, ul. Wielicka 265, 30-663 Cracow, Poland; 2 Department of Pediatrics, University Children’s Hospital, Cracow, Poland

**Keywords:** Multisystem inflammatory syndrome in children, Variants of concern, Severe acute respiratory syndrome-related coronavirus, Incidence of MIS-C, Clinical picture of MIS-C

## Abstract

The purpose of this study is to assess the rate, clinical picture, and management of multisystem inflammatory syndrome in children (MIS-C) during the different COVID-19 variants of concern (VOC) domination periods. This was a retrospective analysis of prospectively collected data. The incidence and clinical picture of MIS-C during the original/Alpha (group 1) and Delta/Omicron (Group 2) variant domination periods were compared. Among 108 eligible patients, 74 (68.5%) were hospitalized during the group 1 domination period, and 34 (31.5%) were hospitalized during the group 2 domination period. The median (Me) patient ages were 76 months (interquartile range [IQR] 35–130) and 73 months (IQR 45–118), and 61% and 65% of patients were male, respectively. There was no significant difference in the presence of positive SARS-CoV 2 antibody test results (IgM or IgG) between the groups (84 vs*.* 90%; *p* = 0.54)*.*No differences between groups were observed in fever duration prior to admission (Me [IQR]: 5 days [3–6] vs. 5 days [4–6]; *p* = 0.26) or the presence of mucocutaneous (95 vs. 100%; *p* = 0.41), circulatory (70.3 vs. 61.8%; *p* = 0.86), neurological (6.8 vs. 2.9%; *p* = 0.662), or gastrointestinal symptoms (84 vs. 79%; *p* = 0.59). Respiratory symptoms were more common in group 2 (70 vs. 91%; *p* = 0.015). The need for intensive care unit admission was similar in both groups (16.2 vs. 17.6%, *p* = 1.0). No deaths occurred in the entire cohort. The studied children were characterized by high C-reactive protein and procalcitonin levels, concentrations of ferritin within normal limits, lymphopenia, moderate hypoalbuminemia, and high B-type natriuretic peptide/brain natriuretic peptide (NT-proBNP) concentrations; however, there were no differences between the groups. Intravenous immunoglobulins were administered as a first-line treatment for almost all patients. There was no significant difference in corticosteroid administration between the groups (87% vs. 74%; *p* = 0.11); however, the summary dose of methylprednisolone was higher in group 2 (Me [IQR]″ 12.6 mg/kg [10.5–17.8] vs. 16.4 mg/kg [13.3–19.5]; *p* = 0.03). The median length of stay was 11 days [IQR]: [9–14] and 10 days [8–12], respectively (*p* = 0.065).

*Conclusion*: The clinical course of MIS-C is similar in subsequent pandemic waves; however, the incidence of MIS-C seems to be decreasing.

**Table:** 

**What is Known:** *• The clinical picture of COVID-19 is evolving. Multisystem inflammatory syndrome in children (MIS-C) is a relatively new serious disease connected with SARS-CoV-2 infection, and in subsequent waves of the pandemic, new cases of the disease have been recorded.*	
**What is New:** *• The clinical picture of MIS-C is not specific, but the course is still severe.* *• The incidence of MIS-C during the different pandemic waves is decreasing and the diagnosis in the period of lower prevalance is challenging.*

**What is Known:**

*• The clinical picture of COVID-19 is evolving. Multisystem inflammatory syndrome in children (MIS-C) is a relatively new serious disease connected with SARS-CoV-2 infection, and in subsequent waves of the pandemic, new cases of the disease have been recorded.*

**What is New:**

*• The clinical picture of MIS-C is not specific, but the course is still severe.*

*• The incidence of MIS-C during the different pandemic waves is decreasing and the diagnosis in the period of lower prevalance is challenging.*

## Introduction

In April 2020, during the COVID-19 global pandemic caused by SARS-CoV-2, a new disease was observed and named multisystem inflammatory syndrome in children (MIS-C) [1]. The first case of MIS-C in Poland was detected in May 2020 [2]. Observations have shown that 2–4 weeks after SARS-CoV-2 infection (mostly asymptomatic or mildly symptomatic patients), immune dysregulation and an increased systemic inflammatory response occur [3]. Therefore, the dominant symptom is fever, but in most patients, mucocutaneous, cardiovascular, gastrointestinal, respiratory, and neurological manifestations are observed. The most common laboratory findings are significantly elevated inflammatory markers, neutrophilia, hypoalbuminemia, hyponatremia, and elevated markers of myocardial damage [2, 4–6]. There is no pathognomonic symptom or marker to confirm the diagnosis.

After 2 years of the pandemic, the clinical picture of COVID-19, modified by subsequent dominant variants of concern (VOCs), has changed and become milder but more transmissible in relation to the entire global population [7, 8], but at the same time, an increasing number of hospitalizations among children was noticed [9, 10]. Initially, MIS-C, which required hospitalization in most cases, was a direct reflection of the wave of COVID-19 with the defined time delay in between waves. After 2 years, despite the high contagiousness of the dominant variants of SARS-CoV-2 and the high incidence of COVID-19 cases, the incidence of MIS-C seems to have significantly decreased [11–13]. There is little data comparing the course of MIS-C depending on the dominance of specific COVID-19 variants. First of all, the data on the last variant of Omicron are very scarce. For this reason, we attempted to compare the clinical course in children with MISC during the period of dominance of the original variants and the period of dominance of the new variants.

## The aim of the study

To assess the rate, clinical picture, and management of MIS-C during the different COVID-19 variants of concern domination periods (including Omicron).

## Materials and methods

### Patient identification and selection

This was a retrospective analysis of prospectively collected data. The study included all patients aged from 0 to 18 years diagnosed with multisystem inflammatory syndrome who had been admitted to the Department of Paediatrics University Children’s Hospital of Cracow between November 1, 2020, and June 30, 2022. MIS-C was diagnosed based on the US Centers for Disease Control and Prevention (CDC) definition [14]. Patients were excluded if any bacterial infection was diagnosed.

For each patient, a defined set of data (demographic characteristics, clinical symptoms, laboratory results, treatment, and outcome) was analyzed. The body mass index (BMI) of the patients was transformed to age- and gender-adjusted percentiles using WHO growth charts [15]. Race and ethnicity data were collected because they are risk factors for severe course of COVID-19 [16] and could similarly affect outcomes in MIS-C [5, 6, 17, 18]. Race categories included White, African American, and others. Ethnicity categories included Hispanic or Latino and non-Hispanic or Latino. The PCR tests were run for all patients before admission. According to Polish directions, the PCR tests for SARS CoV-2 were considered positive if the detection of two or more SARS-CoV-2 genes was confirmed (genes: N, E, S, RdRP, or ORF1ab) [19]. Children included in the analysis were managed according to the recommended protocols by American College of Rheumatology protocols [20, 21] by one experienced medical team (including pediatricians, pediatric pulmonologists, and infectious disease specialists). Intravenous immunoglobulin (IVIG) dosing was 2 gm/kg based on ideal body weight. In the patients with cardiac dysfunction, IVIG dose was divided into 2 days. Low-to-moderate corticosteroid dose was defined as methylprednisolone 1–2 mg/kg/day (if another steroid was used the equivalent methylprednisolone dose was calculated for the analysis). The pulses of steroids were defined as methylprednisolone dose of 10–30 mg/kg/day for 3 to 5 days. The cardiology evaluation was provided by the Cardiology Department. The University Children’s Hospital of Cracow is the only hospital in the region with a pediatrics’ intensive care unit (PICU) and a cardiology department which determined the Department of Paediatrics as the leading unit to diagnose and treat MIS-C throughout the analyzed time. According to the hospital policy, the patients treated with inotropic agents and stable condition were not transferred to PICU (criteria for PICU admission were defined as need for mechanical ventilation or hemodynamic instability). The severe MIS-C was defined as 1. PICU admission or 2. presence of hypotension as defined by systolic and/or diastolic blood pressure below the 5th percentile for sex, age, and height; or significant cardiac dysfunction (decreased left ventricular ejection fraction below 55%; or decreased fractional shortening below 25%) or shock [22]. The study protocol was approved by the Jagiellonian University Medical College Ethical Committee. Written and informed consent was obtained from the parents. The data has not been published before.

**Fig. 1:**
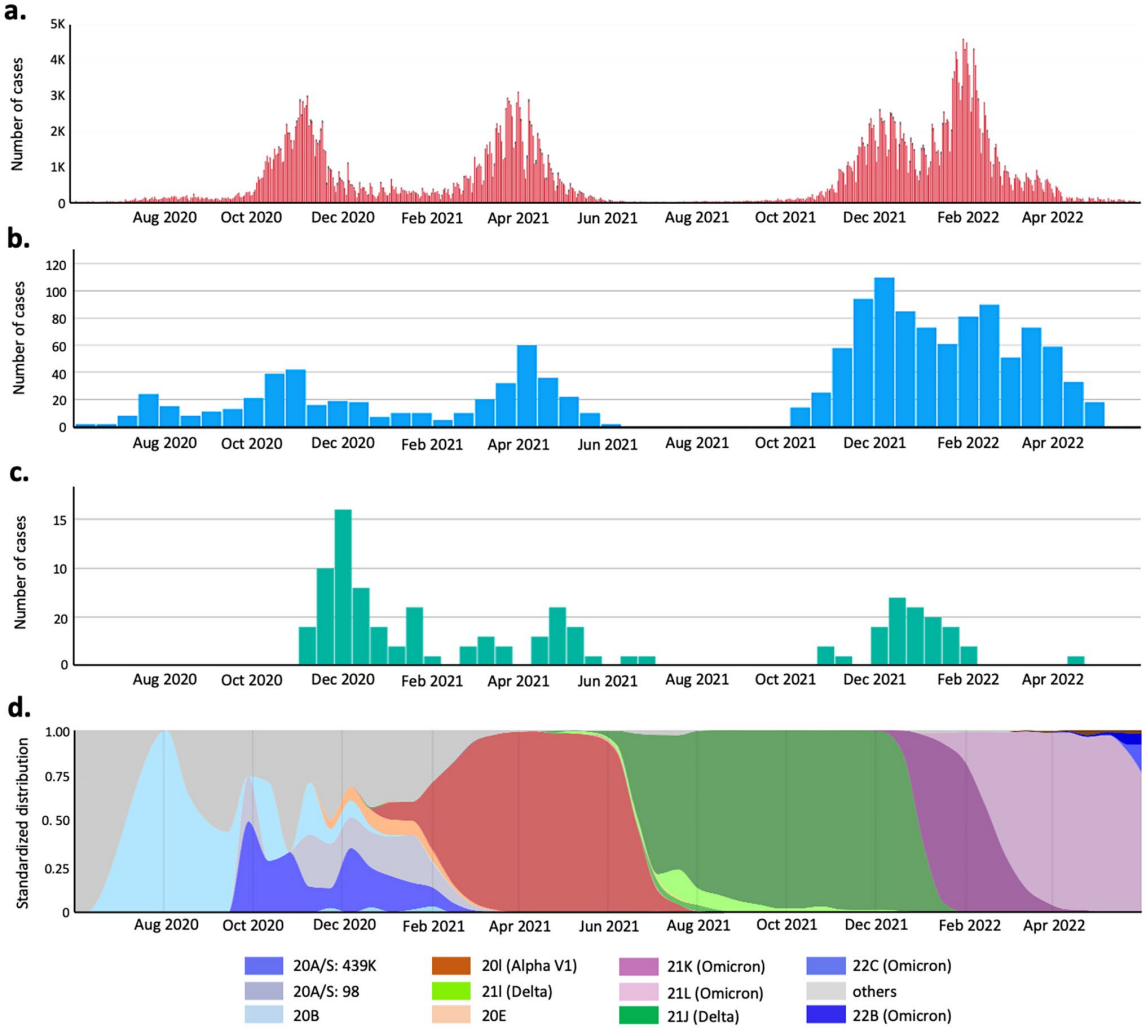
**a** Daily COVID-19 cased in the Malopolska region (based on https://koronawirusunas.pl/u/wojnowe-malopolskie; access date 21.7.2022). **b** Number of children hospitalized due to COVID-19 in the dedicated infectious disease ward in Krakow, Poland (based on Stopyra L, Kowalik A, Stala J, Majchrzak I, Szebla J, Jakosz M, Grzywaczewska K, Kwinta P. Characteristics of Hospitalized Pediatric Patients in the First Five Waves of the COVID-19 Pandemic in a Single Center in Poland-1407 Cases. J Clin Med. 2022 Nov 17;11(22):6806. 10.3390/jcm11226806. PMID: 36,431,283). **c** Number of children hospitalized due to MIS-C in the Department of Pediatrics, Jagiellonian University, Krakow, Poland. **d** The proportion of the total number of COVID-19 sequences over time in Poland (based on www.covariants.org; access date: 21.07.2022)

The number of confirmed COVID-19 cases in the Małopolska region during the study period was downloaded from www.koronawirusunas.pl/u/malopolskie [23], provided by the Polish government. The number of children hospitalized due to COVID-19 was obtained from a dedicated infectious disease unit for the region [10]. The association between SARS-CoV-2 variants and MIS-C cohorts was assumed based on the Polish sequencing database [24], representing the predominant strain of SARS-CoV-2 across time periods (Fig. 1).

### Definitions of groups

Based on the distinct VOC periods of SARS-CoV-2, the following groups were defined:

- Group 1 (original/Alpha variants): patients hospitalized between November 1, 2020, and July 30, 2021—the period of domination of the original and Alpha variants in Poland [24].
- Group 2 (Delta/Omicron variants): patients hospitalized between October 19, 2021, and June 30, 2022—the period of domination of the Delta and Omicron variants [24].

### Statistical methods

First, the Shapiro–Wilk test was used to assess the normality of the distribution of the continuous variables. Because this test indicated that the null hypothesis (normal distribution) should be rejected for most of the analyzed variables, any further data were analyzed using the Mann–Whitney U test. Fisher’s exact test was used to compare the categorical variables. A *p* value of 0.05 or less was considered statistically significant. Statistical analyses were performed using IBM SPSS Statistics v. 27 software (Armonk, NY, USA).

## Results

One hundred eight patients among 110 children admitted with MIS-C suspicion were included in the final analysis. Two MIS-C suspected patients, who developed macrophage activation syndrome and in their further history systemic juvenile idiopathic arthritis was diagnosed, were excluded.

In the domination period of the original/Alpha variants in Poland, 74 patients (68.5%) were hospitalized in our unit due to MIS-C. In the domination period of the Delta/Omicron variants, 34 patients (31.5%) were admitted, of whom 31 (91.2%) were hospitalized during and after 4 weeks of the Delta variant domination period. Only 3 of them were hospitalized during the Omicron variant domination period. All but two patients (98.1%) were non-Hispanic or Latino White. A comparison of selected demographic and clinical variables between the studied groups is presented in Table 1.

**Table 1:** The demographic data and COVID-19 status of the studied groups

Group	Group 1 original/Alpha [*N* = 74]	Group 2 Delta/Omicron [*N* = 34]	*p* value
Male, *n* (%)	45 (61)	22 (65)	0.83**
Age [months], Median (IQR)	76 (35–130)	73 (45–118)	0.82*
Race/ethnicity			
White, *n* [%]	72 (97,29)	34 (100)	1.0***
African American, *n* [%]	1 (1,35)	0	
Hispanic or Latino, *n* [%]	1 (1,35)	0	
Weight [kg], median (IQR)	22 (14–36)	20 (16–33)	0.81*
BMI percentile, median (IQR)	46 (13–70)	42 (8–68)	0.53*
Positive anti-SARS CoV-2 antibodies, *n* [%]	62 [84]	28 [90]	0.54**
Positive SARS CoV-2 PCR test, *n* [%]	11 [15]	0	0.017**
Confirmed SARS CoV-2 infection in the patient’s past history or confirmed SARS CoV-2 infection in the family, *n* [%]	33 [45]	11 [33]	0.3**
Any type of infection in the past 8 weeks before hospitalization, *n* [%]	20 [27]	15 [44]	0.12**
Vaccination against COVID-19, *n* [%]	0	0	

*IQR* interquartile range, *SARS-CoV-2* severe acute respiratory syndrome coronavirus 2, *PCR* polymerase chain reaction

*p value for Mann‒Whitney U test; **p value for Fisher’s exact test; ***p value for chi-square test

There were no significant differences between the groups in terms of age, sex, or body mass index (BMI). The median (Me) patient ages were 76 (interquartile range [IQR] 35–130) and 73 (IQR 45–118) months, and 61% and 65% of the patients were male, respectively. Positive SARS CoV-2 PCR tests were noted only in group 1 (11 patients vs. 0, *p* = 0.017). There was no significant difference in the presence of positive SARS-CoV 2 antibody test results (IgM or IgG) between the groups (84 vs*.* 90%; *p* = 0.54)*.* Only 45% of the patients in group 1 and 33% of those in group 2 had confirmed contact with SARS-CoV-2-infected persons. On the other hand, 27% and 44%, respectively, had a history of any type of infection before 8 weeks of admission. None in both groups were vaccinated against COVID-19.

The number of confirmed COVID-19 cases in the general population in the Małopolska region (Fig. 1a) corresponded to the number of hospitalizations among children due to COVID-19 infection (Fig. 1b) [10]. The hospitalization rate was higher in children in group 2 (Delta/Omicron variants), while the total number of diagnosed MIS-C patients (Fig. 1c) was only one-third of all cases. The proportion of the total number of COVID-19 sequences over time in Poland is presented in Fig. 1d.

The clinical symptoms of MIS-C in the studied groups are presented in Table 2. No differences between groups were observed in fever duration prior to admission (Me [IQR]: 5 [3–6] vs. 5 [4–6] days; *p* = 0.26) or the presence of mucocutaneous (95 vs. 100%; *p* = 0.41), circulatory (70.3 vs. 61.8%; *p* = 0.86), neurological (6.8 vs. 2.9%; *p* = 0.662), or gastrointestinal symptoms (84 vs. 79%; *p* = 0.59). Only respiratory symptoms were more common in group 2 (70 vs. 91%; *p* = 0.015). Hypotension was present in 27 (37% in group 1 and 13 (38.2%) in group 2 (*p* = 1.0). Decreased ejection fraction (EF) was reported in 24 (32.4%) and 8 (24.2) (*p* = 0.5) and coronary arteries abnormalities in 22 (30%) and 5 (15%) (*p* = 0.15), respectively.

**Table 2:** Comparison of the clinical symptoms between the studied groups

Symptoms	Group 1 original/Alpha [*N* = 74]	Group 2 Delta/Omicron [*N* = 34]	*p* value
Fever before admission [days], median (IQR)	5 [3–6]	5 [4–6]	0.26*
Conjunctivitis/rush/mucosal involvement (erythema or fissures, strawberry tongue), *n* [%]	70 [95]	34 [100]	0.41**
Edema (or erythema of the hands or feet), *n* [%]	25 [34]	14 [41]	0.52**
GI symptoms, *n* [%]	62 [84]	27 [79]	0.59**
Respiratory symptoms, *n* [%]	51 [70]	31 [91]	0.015**
Circulatory symptoms, *n* [%]	52 [70.3]	21 [61.8]	0.386**
Systolic or diastolic blood pressure below 5th percentile, *n* [%]	27 [37]	13 [38.2]	1.0**
FS < 25%, *n* [%]	14 [19]	5 [15]	0.79**
EF < 55%, *n* [%]	24 [32.4]	8 [24.2]	0.5**
Abnormal imaging of the coronary arteries, *n* [%]	22 [30]	5 [15]	0.15**
Neurologic symptoms, *n* [%]	5 [6.8]	1 [2.9]	0.662**

Respiratory symptoms: cough, wheezing, dyspnea, auscultatory changes, abnormalities in chest radiography or ultrasound (pleural effusion, consolidations)

Mucocutaneous symptoms: conjunctivitis, skin rash, lip swelling or redness, swollen lymph nodes

Neurological symptoms: headache, diplopia, altered mental status, seizure

Cardiovascular symptoms: tachycardia, hypotension, left ventricular ejection fraction (LVEF) < 55%, coronary dilatation or aneurysm

GI (gastrointestinal) tract symptoms: nausea, vomiting, diarrhoea, abdominal pain

EF ejection fraction, FS fractional shortening

*p value for Mann‒Whitney U test; **p value for Fisher’s exact test

Moreover, the laboratory findings at the beginning of hospitalization were similar in both time periods (Table 3).

**Table 3:** Comparison of selected laboratory results between the studied groups. Data are presented as the median and interquartile range

Group	Group 1 Original/Alpha [*N* = 74]	Group 2 Delta/Omicron [*N* = 34]	*p* value
CRP [mg/l]	155 [65–215]	180 [72–208]	0.43
PCT [ng/ml]	2.44 [1.14–5.61]	1.69 [0.83–4.26]	0.17
Ferritin [ug/l]	250 [136–674]	221 [171–356]	0.39
WBC [× 10^3]	10.3 [7.6–13.1]	10.0 [8.2–13.9]	0.71
% neutrophiles	79 [64–87]	79 [72–85]	0.9
% lymphocytes	11.5 [7–26.2]	13.8 [8.7–19]	0.73
PLT [103/ul]	201 [122–279]	248 [159–354]	0.12
Serum Na [mmol/l]	134 [131–137]	135 [132–136]	0.57
Albumin [g/l]	32.1 [29.4–36.8]	32.6 [29.2–35]	0.55
AST [U/l]	37.5 [31.4–58.2]	34.1 [21.9–43.7]	0.068
ALT [U/l]	26.5 [18–54]	34 [15–61]	0.638
NT proBNP [pg/ml]	2901	2727	0.7

*CRP* C-reactive protein, *NT-proBNP* N-terminal pro brain natriuretic peptide, *PCT* procalcitonin, *WBC* white blood cells, *PLT* platelets

*p* value for Mann‒Whitney U test

The whole cohort was characterized by high C-reactive protein and procalcitonin levels, concentrations of ferritin within normal limits, lymphopenia, moderate hypoalbuminemia, and high B-type natriuretic peptide/brain natriuretic peptide (NT-proBNP) concentrations.

The management and outcomes are presented in Table 4.

**Table 4:** Comparison of management and outcomes between the studied groups

Group	Group 1 Original/Alpha [*N* = 74]	Group 2 Delta/Omicron [*N* = 34]	*p* value
IVIG, *n* [%]	71 [96]	34 [100]	0.55**
Steroids, *n* [%]	64 [87]	25 [74]	0.11**
Steroids—total dose (mg/kg), median (IQR)	12.6 (10.5–17.8)	16.4 (13.3–19.5)	0.03*
Pulses of steroids, *n* [%]	5 [6.8]	2 [5.9]	1.0**
Milrinone, *n* [%]	23 [31]	7 [21]	0.36**
Dopamine, *n* [%]	8 [11]	3 [9]	1.0**
Epinephrine, *n* [%]	6 [8.1]	4 [11.8]	0.72**
Duration of vasoactive support (days), median (IQR)	7 (6–9)	8 (5–12)	0.71*
PICU admission, *n* [%]	12 [16.2]	6 [17.6]	1.0**
PICU LOS (days), median (IQR)	7 (4–9)	6 (3–8)	0.6*
Intubation, *n* [%]	1 [1,35]	2 [5,8]	0.45**
LOS (days), median (IQR)	11 (9–14)	10 (8–12)	0.065*

*IVIG* intravenous immunoglobulin, *LOS* length of stay, *PICU* pediatric intensive care unit

*p value for Mann‒Whitney U test; **p value for Fisher’s exact test

Intravenous immunoglobulins were administered as a first-line treatment for 96% of the patients in group 1 and 100% of the patients in group 2. There was no significant difference in the number of patients treated with corticosteroid low-moderate dose between the groups (87% vs. 74%; *p* = 0.11); however, the summary dose of corticosteroids was higher in group 2 (Me [IQR]″ 12.6 mg/kg [10.5–17.8] vs. 16.4 mg/kg 13.3–19.5]; *p* = 0.03).

The need for intensive care unit admission was similar in both groups (16.2 vs. 17.6%, *p* = 1.0), one of whom from group 1 (1.35%), and two from group 2 (5.8) were mechanically ventilated. None of our patients were treated with ECMO. No deaths occurred in the entire cohort. The median length of stay was 11 (9–14) and 10 (8–12) days, respectively (*p* = 0.065).

There was no difference between the groups in terms of the rate of steroid pulse therapy (6.8% vs. 5.9%; *p* = 1.0). This therapy was administered in seven patients, one of whom was African American and one was White Hispanic or Latino.

## Discussion

After almost 2 years of observation of MIS-C, during the changing of the clinical picture of COVID-19 infection and the popularization of immunization, we observe decreasing rate of MIS-C cases, but the clinical course of the disease remains the same and life threatening. The data including dominant period of Delta and especially Omicron variants are lacking. Proper prompt diagnosis and avoiding mis- and overdiagnosis becomes particularly even more demanding.

In 2020, the World Health Organization (WHO) declared a pandemic of SARS-CoV 2; from that time, except for the original/wild virus, the most important variants of concern (VOCs) identified in Poland were the Alpha (B.1.1.7), Delta (B.1.617.2), and Omicron (B.1.1.529) variants [23].

In the domination period of the original/Alpha variants (September 2020–June 2021) in Poland, there were 2,779,002 cases of confirmed SARS-CoV-2 infections. During this period (group 1), educational establishments (universities, schools, kindergartens, nurseries) were closed. During domination period of the Delta/Omicron variants (September 2021–June 2022), there were 3,124,773 cases [25]. The sanitary restrictions were less severe, and for children, they were practically abolished. From February 2022, for the first time in Poland, antigen tests were widely available, so new infections were confirmed at home and not always registered. Additionally, an upward trend in hospitalization in the group of pediatric patients was observed at this time [10, 11]. It can be concluded that the real incidence rate of COVID-19 during the later period (group 2) was much higher than that of the official statistics. Despite the increasing rate of new COVID-19 infections or COVID-19 reinfection, a significant decrease in MIS-C cases was observed. A similar trend is noted by CDC in the USA [12] or in different studies [11, 13], where the reversal of the proportion between COVID-19 infections and MIS-C is clearly visible. Firstly, it can be connected with different VOC occurrence. Secondly, with an increasing number of children who had COVID-19 at least once, the risk of recurrence of a dysregulated immune response following reinfection with SARS-CoV-2 resulting in MIS-C is still unknown, but seems to be unlikely [26, 27].

Additionally, beginning in May 2021, vaccination against COVID-19 was available for subsequent pediatric age groups (for ages 16–18 years from May 2021, for ages 12–16 years from June 2021, and for ages 5–12 years from December 2021) [28]. Until August 2022 immunization status in Poland was 20% in 5- to 9-year-old children and approximately 55% in 10- to 18-year-old children [29]. The available data confirm that immunizations are not a risk factor for MIS-C [30–33] and there is strong evidence that mRNA immunizations decrease the risk of MIS-C [33]. Our patients also did not receive any vaccination against COVID 19, but MIS-C is possible and should be included in differential diagnosis even in vaccinated patients [32]. On the other hand, rare cases of hyper-inflammatory syndrome with multi-organ involvement following COVID-19 mRNA vaccine with no proof of COVID-19 infection have been described [34].

The demographics were uniform across both groups. Interestingly, the median age of our cohort was lower than those in previous reports from Poland (76 and 73 months compared to 106 months) [35], the UK (98 months) [4], and the USA (104 months) [5]. In both groups, a predominance of males was observed (61% and 65%), which is consistent with previous reports [4, 5, 35]. The majority of patients had laboratory confirmed SARS-CoV-2 infection. Interestingly, only the patients in group 1 had a positive SARS-CoV-2 polymerase chain reaction (PCR) test. It can be assumed that in group 1, the PCR tests were performed in a shorter period from COVID-19 infection or that the virus elimination time was longer at that time. Despite many months of the pandemic, in both groups patients have negative serology, which is consistent with previous report [6, 17, 35]. This observation can be connected with the serological methods limitations [36–39] or possible misdiagnosis. However, the positive serological tests are not obligatory to confirm the diagnosis. Most patients had no medical history of confirmed contact with SARS CoV-2-infected person or any type of infection before 8 weeks of admission. This observation may be related to the fact that COVID-19 infection in children is usually asymptomatic or mild, and tests are rarely ordered [9, 40–42].

The clinical course and severity were similar in both groups. The mucocutaneous, GI, circulatory, and neurological symptoms and laboratory results were consistent with previous reports [4, 5, 35]. The only difference in our group was the higher rate of respiratory symptoms in group 2. PICU admission, the use of catecholamines, the need for intubation, and length of mechanical ventilation were similar in both time periods. Our results differ from other Polish data [35], because the MIS-C course in our cohort was more severe. The main inconsistency is about PICU admission and cardiac insufficiency which were higher in our group. It must be highlighted that our hospital is the largest in the region and includes the PICU, cardiology unit, and cardiac surgery unit. The PICU admission criteria were very strict also. Possibly that is the reason why the data from different reports showed more frequent admissions to the PICU and respiratory support during original/Alpha wave than our data, while the length of hospitalization was shorter [4, 5, 43]. Parag J. et al. retrospectively analyzed patients from Texas Children’s Hospital (Houston) and concluded a milder MIS-C course among patients from the Delta variant domination period. Based on observations, these patients stayed at the hospital for a shorter time and less often needed respiratory or ECMO support. In contrast, no differences in the symptoms or laboratory test results were shown [44]. We did not confirm these observations. In our opinion, the less frequent need for mechanical ventilation or ECMO can be a result of the higher experience of medical personnel in the second period of the pandemic (Delta/Omicron variants), especially for obtaining strict fluid balance and providing bolder and faster administration of steroids. Levy N. prospective study from 12 Israel hospitals suggests MIS-C decreasing incidence and less severe outcome during the Omicron wave of the COVID-19 pandemic. In our study, we have only three patients after the Omicron wave, which is compatible, On the other hand, comparing to our study, in Levy N et al. report the incidence of PICU admission in all three waves was again higher than in our cohort, but the use of vasoactive agents during Alpha and Delta wave was lower [11]. In the study from South Africa no cases of MIS-C were reported after Omicron outbreak. This finding could possibly corroborate to the conclusion that incidence of MIS-C is decreasing, but the observation time period was only 6 weeks from the first case of Omicron detection [13].

It is not established how race/ethnicity is associated with the severity of the disease. In several case studies the predominance of Black, Hispanic, or Latino is described [5, 6, 17, 45]. The predominance of the White race in our cohort could be considered a possible explanation for the milder clinical presentation in Poland than in the other studies. This observation requires further analysis.

Treatment of MIS-C was provided according to the American College of Rheumatology Guidelines published in April 2020 and April 2021 [20, 21]. The first-line treatment was IVIG alone or combined with corticosteroids. Corticosteroids were administered insignificantly less often in group 2, but simultaneously, the dose prescribed in total was higher. It refers to the increasing experience of the clinical team, which allows the administration of steroids to patients in more severe clinical conditions and sets the starting dose at 2 mg/kg methylprednisolone. The need to use the second-line treatment (pulses of intravenous methylprednisolone therapy) was similar in both groups. The retrospective analysis by Ouldali N. et al. showed that combined treatment with methylprednisolone and IVIG versus IVIG alone was associated with a better course of fever in MIS-C patients [46]. Additionally, published from 2022, D. Sofia study by Villacis-Nunez DS. et al. showed that corticosteroid monotherapy can be used as the first-line treatment in patients with mild MIS-C [47]. On the other hand, the international observation cohort study (BATS Consortium) showed no evidence of differences in outcomes between treatment with corticosteroids or IVIG as single agents or between the single-agent and dual-agent primary treatments [48]. These observations required further research.

Our study has some limitations. This was a single-center study. The sequencing data for SARS-CoV-2 variants were estimated from the central Polish laboratory; we did not sequence distinct variants in our laboratory. The CDC guidelines were used for the identification of our cohort, but the overlapping and nondifferentiating clinical presentation of MIS-C, acute COVID-19, and other acute infectious diseases can interfere with the clinical judgement [49].

The comparison of the course of MIS-C during different pandemic waves in the group of patients treated by one experienced team using the uniform diagnosis criteria, the uniform laboratory test protocol, the uniform treatment approach, and cooperating with one dedicated consulting clinicians reliably shows that clinical picture of MIS-C is still the same, only the rate is decreasing.

## Conclusion

Despite changing the clinical picture of COVID-19, the clinical course of MIS-C remains the same. However, the MIS-C prevalence is decreasing. MIS-C remains a serious, life-threatening condition that has no pathognomonic symptoms or markers. The diagnosis in the period of lower prevalence is challenging.

## Abbreviations

BMI: Body mass index
CDC: Centers for Disease Control and Prevention
COVID-19: Coronavirus disease 2019
CRP: C reactive protein
ECMO: Extracorporeal membrane oxygenation
GI: Gastrointestinal
ICU: Intensive care unit
IQR: Interquartile range
IVIG: Intravenous immunoglobulin
LOS: Length of stay
MIS-C: Multisystem inflammatory syndrome in children
NT-proBNP: B-type natriuretic peptide/brain natriuretic peptide
PCR: Polymerase chain reaction
PCT: Procalcitonin
SARS-CoV-2: Severe acute respiratory syndrome coronavirus 2
VOC: Variants of concern
WHO: World Health Organization
